# It takes a village: an ethnographic study on how undergraduate medical students use each other to learn clinical reasoning in the workplace

**DOI:** 10.1007/s10459-024-10404-5

**Published:** 2025-02-10

**Authors:** Larissa I. A. Ruczynski, Marjolein H. J. van de Pol, Shiba Hashmi, Erwin J. H. Vos, Cornelia R. M. G. Fluit, Bas J. J. W. Schouwenberg

**Affiliations:** 1 Research on Learning and Education, Radboudumc Health Academy, Radboud University Medical Center, Gerard Van Swietenlaan 2 (Route 51), 6525 GB Nijmegen, The Netherlands; 2 Department of Primary and Community Care, Radboud University Medical Center Nijmegen, Nijmegen, The Netherlands; 3 Department of Internal Medicine, Slingeland Hospital, Doetinchem, The Netherlands; 4 Department of Emergency Medicine and Intensive Care Medicine, Elkerliek Hospital, Helmond, The Netherlands; 5 Research on Learning and Education, Radboudumc Health Academy, Radboud University Medical Center Nijmegen, Nijmegen, The Netherlands; 6 Department of Pharmacy, Division Pharmacology-Toxicology, Radboud University Medical Center Nijmegen, Nijmegen, The Netherlands; 7 Department of Internal Medicine, Radboud University Medical Center Nijmegen, Nijmegen, The Netherlands

**Keywords:** Clinical reasoning, Clinical decision making, Peer-assisted learning, Medical education, Workplace learning

## Abstract

When students learn with–and from–other students, it is called peer-assisted learning (PAL). How undergraduate medical students use their peers for their clinical-reasoning learning process remains unclear, although literature suggests that it is a promising learning strategy at this stage. This research therefore explores the question: ‘How is PAL manifested in the clinical learning environment of undergraduate medical students with regard to developing clinical-reasoning skills?’. A constructivist paradigm with a sociocultural theoretical framework was adopted for this research, using PAL and workplace learning as theoretical background. Focused ethnography and a combination of template and open coding was used to gather and analyze qualitative data. Twenty semi-structured interviews were conducted with nine students, four residents, and seven clinical supervisors. A total number of 31.5 h were used for five clinical observations. Following categories were used to describe the data: (1) the role of PAL in the clinical-reasoning learning practice, in which PAL-theory was placed alongside clinical practice, (2) the role of different actors during PAL and (3) the PAL environment, in which contextual factors have been scrutinized. Students deploy various categories of PAL to advance their clinical-reasoning skills, although they were largely unaware of these processes, and facilitation of PAL is not consistently provided. Three topics of discussion are identified that need to be acknowledged: (1) the design of a PAL environment that is conducive to collaborative learning, (2) the shifting roles of peers when they enter clinical practice, and (3) the individualistic tendencies of students that can hamper PAL. Future research could focus on stimulating and facilitating PAL among the next generation of students and integrating PAL into the clinical practice workflow.

**Supplementary Information:**

The online version contains supplementary material available at 10.1007/s10459-024-10404-5.

## Introduction

The development of clinical-reasoning skills is a well-researched topic in the Health Professions Education literature (Yazdani & Hoseini Abardeh, 2019; Young et al., 2020). Throughout undergraduate clinical clerkships, students use all kinds of resources for their own clinical-reasoning learning process (Anakin et al., 2019; Ruczynski et al., 2022; Steven et al., 2014). Previous research demonstrated that this not only includes patients, supervisors and clinical contexts but also other students (Ruczynski et al., 2022). Whereas peer-assisted learning (PAL) is widely used in the preclinical teaching of medical students, recent literature states that PAL is particularly useful to students in the clinical stages of their education where the clinical-reasoning learning process accelerates (Brierley et al., 2022; Ruczynski et al., 2022). Nevertheless, there is a notable decline in the extent to which students use each other for their own learning processes during clinical clerkships compared to the preclinical years (Bransen et al., 2022), leading to the underutilization of peers in the clinical-reasoning learning process.

When students learn with–and from–other students, it is called peer-learning or peer-assisted learning (PAL) (Topping & Ehly, 1998). There are different formal definitions of PAL, for example the one from Lincoln and McAllister, who defined it as *‘to get knowledge through study, experience, observation or teaching of an equal’ *(Lincoln & McAllister, 1993). Another acknowledged definition of PAL comes from Topping’s book wherein PAL was described as *‘people from similar social groups, who are not professional teachers, helping each other to learn and by doing so, learning themselves’* (Topping & Ehly, 1998). Various categories have been identified under the umbrella of PAL (Box 1), of which peer teaching (Topping, 2005; Topping & Ehly, 2001), peer collaboration (Ladyshewsky, 2000; Topping & Ehly, 1998), peer modelling (Raat et al., 2010; Topping & Ehly, 2001), and peer assessment(Topping 1998, 2005) are the best known.

Numerous positive effects of PAL have been found, such as an appreciation of lifelong learning and cognitive development in general(Tai et al., 2016), but also gaining facilitation skills, the development of confidence and feedback techniques for peer tutors specifically (Burgess et al., 2014). Multiple other profits have been hypothesized, like reflection skills, deeper learning, professional identity formation and self-directed learning (Tai et al., 2016; Topping & Ehly, 2001). These are, of course, all desirable skills and competencies for future healthcare workers. PAL has also demonstrated its efficacy in improving academic performance (Brierley et al., 2022), which raises the question of how this applies to clinical reasoning in clinical practice. Challenges for PAL stem from the historically individualistic approach in medicine and medical education (Bleakley, 2020), and how attention to PAL should balance with the focus on individual performance. It has also been criticized for potential negative social impacts (Raat et al., 2010; Ruczynski et al., 2022; Tai et al., 2016) and quality has been questioned when PAL is used for formal peer assessment and feedback (Burgess et al., 2014; Tai et al., 2016). A deeper understanding of underlying mechanisms could help to use PAL more deliberately for learning clinical reasoning.

Over the past decade, there has been an increasing focus on undergraduate students and their perspectives on the clinical-reasoning learning process, particularly during clinical clerkships (Anakin et al., 2019; Ruczynski et al., 2022; Steven et al., 2014). Within this body of knowledge, some research addresses PAL during undergraduate clerkships (Tai et al., 2014, 2016, 2017). However, the impact of context within PAL that is focused on the clinical-reasoning learning process during that specific learning phase is given very little attention. This research therefore explores the following question: How is peer-assisted learning manifested in the clinical learning environment of undergraduate medical students with regard to developing clinical-reasoning skills? To answer this question, two sub-questions were formulated: (1) Which categories of PAL are identifiable within the clinical learning context of undergraduate students developing clinical-reasoning skills? And (2) how do different factors in this context influence PAL in the workplace for the development of clinical-reasoning skills by students? Answers on these research questions can help us support students’ (clinical-reasoning) learning practice.

## Methods

### Theoretical framework

This research was conducted under a constructivist paradigm and using Vygotsky’s sociocultural theoretical framework to understand learning. This framework posits that individuals learn through interactions with their social environment, tools and cultural practices. Our theoretical framework leans heavily on PAL (Box 1), but since this research is conducted at the workplace, the principles of workplace learning were used for the interpretation of the data as well. During clerkships, learning occurs primarily in the clinical workplace (Anakin et al., 2019; Ruczynski et al., 2022; Steven et al., 2014). Mechanisms that are important for learning in the workplace include learning through supervised participation, role modeling, and reflection in action (Wiese et al., 2018).

In this research, we specifically looked at learning clinical reasoning in the medical profession. Clinical reasoning is commonly referred to as the analytical process in which a physician works on a patient’s problem (Young et al., 2018). During this process, the patient’s history, physical examination, and diagnostics are used to form a possible diagnosis and treatment plan. Over the years, clinical reasoning has evolved beyond merely consisting of separate skills that can be acquired. It has become a multi-faceted construct where the complexity of clinical practice is a recurrent aspect (Young et al., 2018). Defining it can pose a challenge, but within the scope of this research, we adopt the most inclusive definition available, where *‘anything that contributed to the final outcome of reasoning is included and permissible as a component of clinical reasoning’* (Young et al., 2018). Examples include inter or intra-professional work relationships, contextual or personal factors, or doctor-patient relationships.

**Box 1:** Categories of peer-assisted learning (PAL)

Various categories under the umbrella of PAL can be discerned, and these will be used as a theoretical background. All of these categories can be seen as deriving from Piaget’s and Vygotsky’s belief that interaction is crucial to learning (Topping, 2005). Within the context of this interaction between peers, four categories of PAL can be distinguished. The most common category is *peer teaching*, based on Vygotsky’s *zone of proximal development* (1978), where a more knowledgeable tutor pulls the student–or tutee–into the boundary of their knowledge where the tutee will start developing (Topping, 2005; Topping & Ehly, 2001). Another well-known category of PAL is *peer collaboration,* which can be linked to cooperative learning, since herewith, students work together equally towards a common goal (Ladyshewsky, 2000; Topping & Ehly, 1998). A third category of PAL is *peer mentoring,* which encompasses students’ relationships – mostly informal—with other students, in which they offer each other peer discussion, nurturing, sharing, encouragement, and support (Henning et al., 2008; Topping, 2005). Peer mentoring overlaps with *peer coaching*, but as a separate category, peer coaching is explained as peers who provide consultative assistance to each other through the use of theory, observation, demonstration, practice and feedback (Ladyshewsky, 2000). Peer coaching also facilitates the transfer of knowledge, skills and attitudes achieved through training (Ladyshewsky, 2000) Students engaging in PAL are constantly near one another. It is almost inevitable that they will compare themselves to their peers and form judgments of themselves and those peers. In this context, three other categories of PAL can be distinguished. The first is *peer modeling*, where students see peers as competent – but not necessarily perfect—‘models’ to which they can compare their own performance, in order to gain insight in their own capabilities, limitations, opportunities and threats (Raat et al., 2010; Topping & Ehly, 2001). The second category in this matter is *peer monitoring,* which refers to students keeping an eye on their peers’ learning behavior and whether their peers are going through appropriate and effective processes and procedures of learning (Brown et al., 1999; Topping & Ehly, 2001). The third category is *peer assessment*, which encompasses both peers *‘quantitatively evaluating the products or outcomes of learning of others in the group’* and *‘giving formative and qualitative feedback to peers’* (Topping, 1998, 2005)	

### Focused Ethnography

Employing a focused ethnographic (FE) approach in this research, we gathered data through observations and interviews (Andreassen et al., 2020; Higginbottom et al., 2013; Rashid et al., 2019). Traditional ethnography is characterized by spending extended periods of time in the field and immersing oneself in a research setting, its societies, and cultures to collect comprehensive information on social structures and behaviors (Andreassen et al., 2020). In comparison, FE is a suitable methodology for examining collective experiences related to a specific, limited, and predetermined phenomenon within subcultures of complex societies (Rashid et al., 2019). In our case, we investigated the manifestation of PAL in the clinical-reasoning learning process (specific, limited, predetermined phenomenon) of medical students during clinical clerkships (subculture) within the broader clinical health environment (complex society). This targeted approach often allows data collection in less time than traditional ethnography.

We opted for semi-structured individual interviews, as that method aligns with our objective to explore the profound personal viewpoints and experiences of each participant (Dicicco-Bloom & Crabtree, 2006). By selecting this approach, we aimed to avoid any potential influence among participants. We also sought to provide a socially safe environment, acknowledging the presence of hierarchical dependency and power dynamics within the diverse group (Looman et al., 2022). By starting with individual interviews, we gathered more information about the phenomenon under investigation (PAL) in this FE that helped us specify what to focus on and in which contexts to organize the clinical observations.

Additionally, we used clinical observations of undergraduate medical students at their clerkship to better understand these peer-to-peer moments. Our belief and previous experience were that certain learning and teaching moments are often not recognized by participants. Drawing upon our desk review of the various categories of PAL (Box 1), we hypothesized that more additional activities or phenomena could be occurring than participants were able to tell us during interviews. This follows the theories of informal learning in workplace where learning is partly implicit, unintended, opportunistic, and unstructured (Eraut * & M., 2004; Tynjälä, 2008).

### Research context

This research was conducted at the Radboud University Medical Center in Nijmegen, The Netherlands, and three affiliated teaching hospitals (Canisius Wilhelmina Hospital Nijmegen, Elisabeth-Tweesteden Hospital Tilburg and Jeroen Bosch Hospital ‘s-Hertogenbosch). The Master’s program covers a duration of three years, during which students partake in ten clinical rotations. The first seven rotations follow a predetermined sequence, while the final year can be customized by students based on their personal preferences. Preceding and following each rotation, a formal teaching program is organized wherein students prepare for and reflect on their experiences in practice. A new group of 28 students starts the Master’s program each month.

### Inclusion

Students of the Master’s curriculum of Medicine in the Netherlands were eligible for inclusion if they had their clerkships at the Radboud University Medical Center or an affiliated teaching hospital between October and December 2022. Residents within the same hospitals were also recruited for the interviews. Additionally, supervisors of different clerkships within our curriculum were included if they possessed firsthand experience in supervising groups of students during their clerkship at their respective department. These supervisors could be medical specialists, clerkship coordinators or both. All candidates were recruited through e-mail. Next to voluntary enrollment, purposive sampling was used for the inclusion of participants to ensure broad perspectives and to purposefully organize clinical observations (Moser & Korstjens, 2018). The inclusion of participants ended when the research group determined that they had collected sufficient data to answer the research question and facilitate data adequacy (Erickson, 1985; Varpio et al., 2017).

### Reflexivity

The research group brings broad medical expertise, spanning primary care, emergency care, internal medicine, and elderly care. All researchers are active in the medical curriculum: as program director (MvdP), educator (MvdP, CF, BS, LR), clerkship coordinator (BS) and clinical supervisor (MvdP, BS). Due to potential power dynamics stemming from MvdP’s role as a program director and BS’s prominence as an educator, they had no contact with participants during the research process. Two medical students, SH and EV, participated concurrently as research assistants for their scientific internships under the supervision of LR and CF. This could have led to socially desirable attitudes in discussions about the data during the period when they were part of the research group. However, we explicitly informed them that their contributions would not affect their final grade, and separate meetings were organized between SH, EV and LR to create an environment where they could freely express their opinions. Both were in the final stage of their undergraduate education and had just completed two and a half years of clerkships. Because of this, SH and EV both were acquaintances of some of the student-participants. Some of the supervisors were known to LR because of the shared educational roles and interests, but none of them have worked together with LR before. There were some epistemological differences within the research group, which enriched discussions during this research until, ultimately, consensus was reached on how to move forward.

### Data collection

The research was conducted between October and December 2022. The interview guide for the individual interviews was prepared by LR and SH (see Appendix 1) and subsequently discussed in the research team (Dicicco-Bloom & Crabtree, 2006; Moser & Korstjens, 2018). The PAL-categories (see Box 1), clinical reasoning, and predetermined examples of PAL-encounters were used as sensitizing concepts, providing a general sense of direction for this research (Bowen, 2006). The guide contained questions on peer-to-peer contact moments in practice, and people involved and their roles. There was ample room to explore other statements contributed by participants. Interviews were done by either LR alone (3x), LR with SH (15x) or SH with EV (2x). They were audio recorded and transcribed verbatim by LR, SH and EV, after which the audio files were deleted. Participants were pseudo-anonymized for confidentiality purposes. Only the head researcher (LR) was able to connect data to individual participants.

After the individual interviews, clinical observations commenced with other participants than previously interviewed. They were done by both LR and EV, who participated as passive observers in the field. They independently made hand-written fieldnotes using an observation guide based on *Wolfinger’s* work (Wolfinger, 2002), wherein the PAL categories (Box 1) and clinical reasoning were used as sensitizing concepts (see Appendix 2). Within these fieldnotes, participants were fully anonymized, being given a codename from the start. When necessary, LR and EV split up as students went their separate ways to incorporate varied situations in the data. Short, unstructured interviews were held during and after observation to gain more insight into what was observed, as well as for member checking and triangulation purposes (Rashid et al., 2019). These interviews were recorded and transcribed verbatim afterwards. The hand-written fieldnotes were digitally processed by LR and EV immediately and individually after the observations.

### Data analysis

**Fig. 1:**
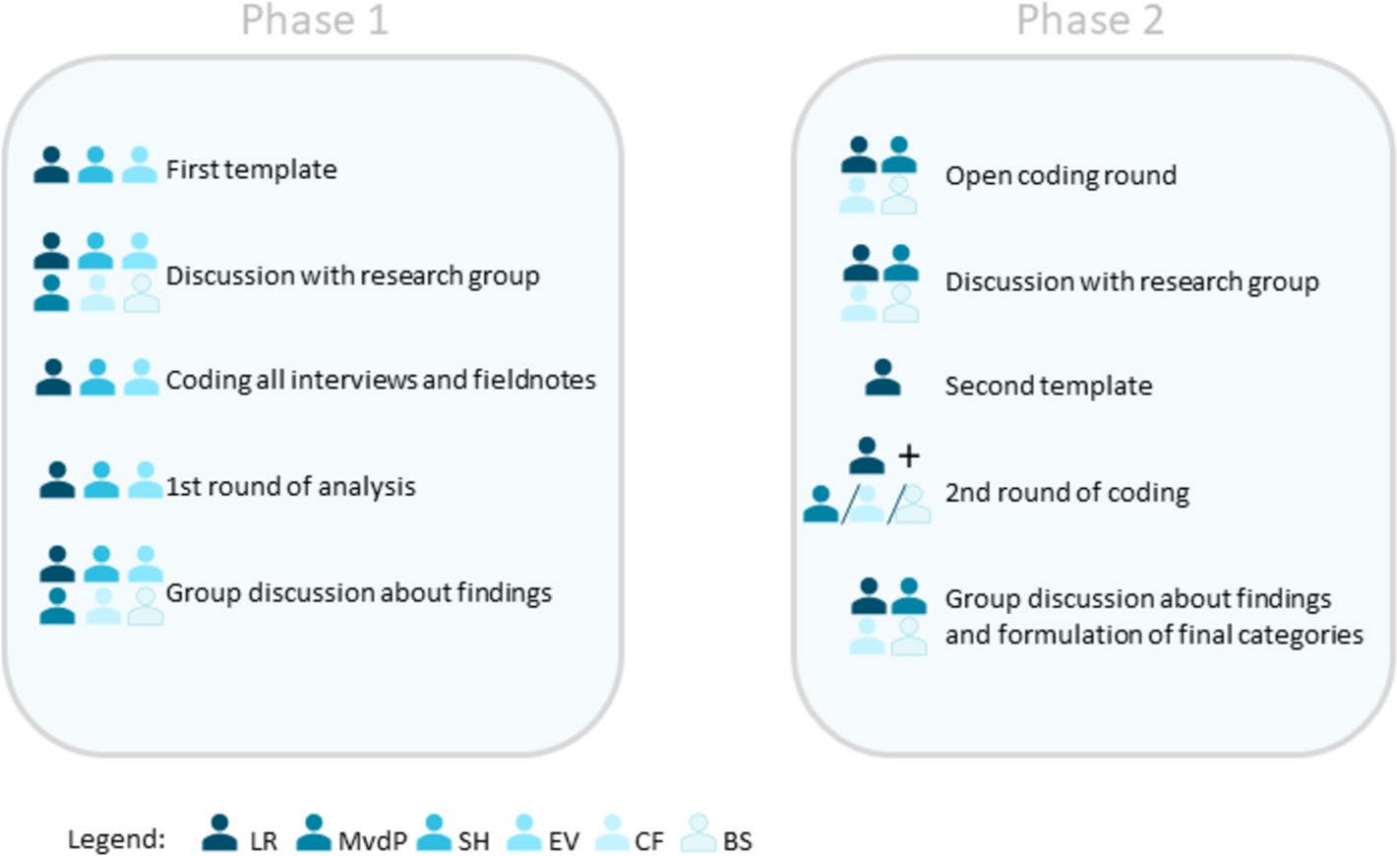
Data analysis research activities

Interview transcripts and fieldnotes were coded using a combination of template analysis and open coding (Braun & Clarke, 2006; Kiger & Varpio, 2020; King, 2004) in Atlas.ti. Analysis was done in two phases of which an overview can be found in Fig. 1. The initial coding scheme, based on the interview and observation guides, was developed after becoming familiar with the data (LR, SH, EV). Main themes in this initial template were (1) PAL-categories, (2) context of PAL, (3) outcomes of PAL, (4) missed opportunities for PAL and specifically for the fieldnotes also (5) possible forms of bias. The various categories of PAL, as described in Box 1, were used as subthemes withing the ‘PAL-categories’ theme. The template was discussed among the research group (LR, SH, EV, CF, MvdP, BS) prior to the head researcher (LR) and a research assistant (SH, EV) coding all interviews and fieldnotes. An initial, deductive, round of analysis was conducted by LR, SH and EV and the findings were subsequently discussed with the other researchers (CF, MvdP, BS). The research assistants (SH and EV) completed their scientific internships after phase 1 and were from that point no longer involved until the writing process.

After the first round, a second round of analysis was performed to reach sufficient depth to answer our research questions. Following the aforementioned group discussion, modifications were made to the template, and a second round of coding was initiated. By using PAL-categories (Box 1) as codes during the first round, a judgment had to be made before applying the code to a portion of text, as the various PAL-categories were rarely seen in practice exactly as the theory describes. This did not align with the intention to stay close to the data, so during a small inductive round, open coding was employed to formulate subthemes within the ‘PAL events’ theme. Three subthemes were identified from the open codes (see Appendix 3): (1) the role of others in PAL, (2) the role of oneself in PAL and (3) student interactions in PAL. With these new subthemes, a final round was initiated wherein major themes in the template were (1) PAL-events (with the new subthemes), (2) Actors, (3) Context, (4) Individual factors, (5) Suggestions for improvement and (6) Clinical reasoning. All interviews and fieldnotes were then independently coded by at least two researchers: the head researcher (LR) with CF, MvdP, or BS. In group sessions, final categories were formed through ongoing analysis and discussion with the research group (LR, CF, MvdP, BS), utilizing different forms of PAL as a framework for the interpretation and presentation of results. Further modifications were done throughout the writing process.

### Ethical review

Ethical approval was granted by the ethical review board of the Dutch Association for Medical Education (the NVMO), case number 2022.1.10.

## Results

Twenty semi-structured individual interviews were conducted with nine students, four residents and seven clinical supervisors. Spread across five clinical observations, a total number of 31.5 h were used to observe a total of 26 undergraduate students at the Internal Medicine, Neurology, Surgery, Ophthalmology, and the Emergency department. During group discussions with the whole research team, we ultimately formulated the following three categories that align with our two research subquestions:

1. The manifestation of PAL in the clinical-reasoning learning practice
2. The PAL environment
3. The role of different actors in the PAL-context

Findings are marked as either coming from interview (I) or observation (O) data or have no mark if substantiated by both. Additional quotations, marked Q# in text, can be found in Table 1.

**Table 1:** Quotations *(marked by Q# in main text; M# indicates the Master’s year)*

Q1	Um...well, it started with me. I opened the patient's case, and at first, I tried to handle it myself. But I knew that I found that topic very difficult. Then, [same-level peer] was sitting next to me, kind of observing, and he suggested, 'You know, there's a really useful section in UpToDate with a lot of information.' So, he gave me that tip, and I started looking at it, which helped a lot at first. But I still found it quite challenging, and then he said, 'I'll just take a look with you,' and we started looking up things together. Like what to consider for different diseases, what to include in the differential diagnosis and what findings you would see with those different diagnoses. He wasn't necessarily asking me questions because he didn't know everything either, but he enjoyed looking things up with me. [...] And I told him what my supervisor had said, and I could explain more because my supervisor had told me. He liked hearing that feedback. So initially, he probably had more knowledge than me because he was interested in this topic, and I found it very difficult, but after seeing the patient, I could tell him more about certain aspects. (M2 student)
Q2	I learn by seeing how things can be done differently. Um...yes, for example, during a family conversation or how to call a supervisor. So, by observing someone I think, 'Oh, I wouldn't do it that way'. When listening to such a conversation as an outsider, I can better observe how things can or cannot be done. (M2 student)
Q3	They share emotions: 'I feel guilty when I see patients in pain, knowing that you still have to proceed.' They reflect: 'You have to be firm during the physical examination; it just has to be done,' and 'I feel this way as a student now, but later as a physician, it will still have to be done.' (observation note of M1 students)
Q4	Um...because I always want to do it myself. I see it as something embarrassing if I have to ask someone else for help. That's one thing. But maybe also because of the competition, that someone else would know that I don't know. (M3 student)
Q5	When you are preparing for your outpatient clinic patient, you have to think ahead about what questions you want to ask, what specific diseases you are considering. Sometimes I'm not sure if I should do any additional type of physical examination, so I ask, 'What would you do in this situation?' Or if I'm unsure whether a particular differential diagnosis fits with this story, I discuss it with other students, so they can think along with me. […] Usually, you also share stories of patients you have seen, and I find it very interesting to hear about what symptoms someone experiences or what symptoms fit with a certain disease. I think that's also a way of learning from each other. So, you learn from each other during preparation, during the consultation, where you exchange stories, and afterwards, from any feedback a specialist might have given me. (M3 student)
Q6	Yes, I found [hearing peers answering questions] very helpful, especially because based on their answers, I could get an idea of their knowledge and see where I still need to improve my clinical reasoning. So, when they gave answers that I hadn't even considered, it stuck with me, and I think that's beneficial for improving my clinical reasoning. […] Hmm, yes, because it rings a bell. Especially when they give answers that make you think, 'I hadn't thought of that,' you end up delving deeper into that topic than you did before. So, in that sense, it does have an impact on my study behavior because I go more in-depth on a particular topic. (M1 student after observation)
Q7	You can also learn from [observing peers], not just about what you don't know, but sometimes how others do things differently. You think, 'Oh, that's also useful, I'll adopt that'. [...] Um...yes, I think so...with differential diagnoses, how you formulate them, when you see how someone else does it and you think, 'Oh, that's also a handy way to categorize things.' (M2 student)
Q8	But I once had an experience at the ward where, during the heart auscultation, I heard a mechanical heart valve, and I found that quite remarkable, so I told my fellow student, 'Wow, I heard such and such, maybe you’d like to listen to it, too.' (M2 student)
Q9	Student 2 is talking about the patient she is currently preparing for the outpatient clinic. Student 1 suggests that she should include a proposal to take a blood sample in her plan, so she can come up with a smart idea when discussing with the supervisor. Student 2 confirms that she has indeed done that. Student 1 adds that she could also ask for an assessment of clinical skills (OSCE) related to it. Student 2 seems grateful for these suggestions and continues their work. (observation note of M1 students)
Q10	I think it's just more comfortable when you have a student meeting room with only fellow students. You can gossip a little *laughs* and discuss things that are on your mind. Because otherwise, well...ultimately, the residents are the ones who will evaluate you, and it's nice to have a separate space to vent and talk with your peers. So, yes, I think having a dedicated student meeting room is more pleasant than a mixed one. (M3 student)
Q11	It also must come from the student's own curiosity, if I may say so. You can endlessly arrange everything for them, but I think that takes away some of the creativity that a professional should have to find their own path and think, “How do I ensure that I learn what I need to learn?” If you institutionalize everything, you take that away, and I think it can actually become stifling. You must be a bit cautious with that. So, you need to give them space, and they have that space with us if they want it. I think there’s actually quite a lot of space. They have few responsibilities, so… well… to then institutionalize that as well, to formalize it in structured ways… I’m not sure about that. (supervisor)
Q12	The hierarchy is that you start with the fellow student. And if you can't figure it out with them, then you ask the resident, because you want to be sure that you have filtered out the simplest questions with the students. And if you still can't find the answer, you think, 'Oh okay, if no one here knows, then I'll ask the next person'. (M3 student)
Q13	I think I consider information from someone who holds a higher position and has studied longer as more likely to be true than something that comes from someone in the same position as me, so to speak. [...] I do think that some physicians are not very good at realizing that not everyone thinks the same way they do, which can make them a bit hasty. So in that case, it's probably nicer to learn from medical interns who can empathize better with how you think. (M2 student)
Q14	A specialist joins the conversation, starting with a compliment and affirmation of the reasoning process so far. He asks a follow-up question to the students, and [student 5] responds. The specialist then poses another question and leaves it with the students as he steps out for a quick phone call. As soon as he leaves, [student 2] shares with the group that she doesn’t know the answer. The group offers suggestions. The specialist returns to the room a few seconds later, and [student 2] answers the question, to which the specialist adds further information. (observation note from formal education at the workplace of M1 students)
Q15	I feel more at ease when a specialist isn’t present because you quickly get the sense that you’re being evaluated—and that’s true, since you’re also being assessed on things like how your presentation goes. So, you’re very aware of that. For example, during my presentation, when the supervisor stepped out for a moment, I thought, “Oh no, I don’t actually know the answer to the question he asked right before he left.” So yeah, you do change a bit, and you… I’m more like, “Okay, how am I going to handle this?”, with the other students. […] From my experience so far, medical specialists want you to be confident. If you don’t know the answer, they still expect you to come up with something. Just saying you don’t know is usually dismissed, or at the very least, they’ll say, “Yes, but at least say something.” Among ourselves as students, we all have moments when we don’t know something, and then it’s easy to just admit it. You don’t have to start talking aimlessly, so to speak. (M1 student after observation Q13)
Q16	And what also yields results is that a resident or supervisor becomes more of a manager of the learning process rather than the one executing the learning process, so that would also save time. (supervisor)
Q17	What I usually did was to keep asking open questions that [students] have to respond to, and especially to encourage them to respond to each other. Not to me like 'what do you think about that?' and then I get an answer back, and I have to pass the ball to someone else – but rather, try to see if you can pass that ball to someone else through your questions. So instead of saying, 'what do you think about it?' try asking, 'what would you have done, or what do you think about that plan?' And with that, you stimulate them to respond to each other, and then you hope to get more interaction between them than just between me and them. (resident)

### The manifestation of PAL in the clinical-reasoning learning practice

Despite not always being explicitly acknowledged, students actively engage in various forms of PAL activities during their clerkships. They collaborate during the preparatory phase of patient encounters, discussing history taking, physical examination, differential diagnosis, and management plans. After the encounter, students share the outcomes with their peers. All their PAL activities can be classified into two categories: peer-to-peer interaction and peer-to-peer relationship/judgment (Box 1).

### Peer-to-peer interaction

#### Peer teaching

Peer teaching is the most present form of PAL in this research. Prominent instances include peer-teaching moments where students present patient cases and explain diseases to their peers. Students also engage in question-and-answer interactions, with one acting as the tutor (presenter or question-answerer) and the other as the tutee (audience or questioner) (Q1). These situations may occur spontaneously or be planned. Notably, we observed that the roles of tutor and tutee can swiftly interchange within a single moment, resulting in a more dynamic peer-teaching scenario (O). For instance, during sparring sessions, students rapidly transition between tutor and tutee roles, reinforcing each other's ideas. Additionally, passive tutees (third-party observers) learn from the peer-to-peer or peer-supervisor exchange (Q2).

#### Peer collaboration

Students engage in collaborative activities within the workplace. They are often made responsible for seeking answers to questions, particularly related to clinical reasoning, that arise from workplace discussions (I). At times, they are required to coordinate with each other to create their own clerkship schedules. Spontaneous peer collaboration frequently occurs when a student faces a pressing question regarding a patient case and mobilizes their peers to collectively construct an answer (O).

#### Peer mentoring

Student groups rely on cohesive bonds based on trust, affirmation, recognition, support, and empowerment (Q3) (I). The absence of these factors can lead to competitive or unsafe learning environments, negatively impacting peer learning (Q4) (I).

#### Peer coaching

Students derive significant learning from sharing their experiences with each other, making it a crucial aspect of their learning process. Through discussions on their interactions and communication with patients, feedback received from supervisors, and by sharing mistakes and challenges with each other, students gain deeper insights into the clinical-reasoning process (Q5). They often provide tips, explanations, and assistance to one another, which enables them to learn about illness scripts that may not have been personally encountered or might not arise during the current clerkship (I). Informal peer coaching predominantly takes the form of anecdotal storytelling, with students often lacking knowledge of appropriate probing questions to facilitate in-depth conversations (O).

### Peer-to-peer relationship/judgment

#### Peer modeling

Students constantly compare themselves to their peers during clerkships. Through discussions about their own work, students assess their standing in comparison to their peers, identify areas that require attention, and determine their level of knowledge (Q6). Additionally, students observe how their peers handle situations that they themselves find familiar. Peers act as explicit role models by demonstrating their approaches, such as history taking, physical examination, or utilization of computer resources for clinical reasoning. This comparison process yields one of three outcomes (I): (1) students modify their learning behavior, (2) they affirm that their current approach is sufficient and continue as before, or (3) they acknowledge differences without assigning value judgments or the need to change anything (Q7).

#### Peer monitoring

While some students may experience competition, most are willing to support each other in achieving their aspirations as physicians. They alert peers to interesting patients (Q8) and learning opportunities, collaboratively plan learning activities, and actively share information (Q9). However, some participants report instances where students predominantly work independently during their clerkships (I).

#### Peer assessment

The least practiced form of PAL was peer assessment. Students appreciate the absence of formal peer assessment to maintain trust and a safe learning environment (I). However, both students and supervisors recognize the potential for formative peer feedback, as supervisors have limited time and should not be required to evaluate every situation or skill (I).

### The PAL environment

PAL takes place in varied environments shaped by multiple influencing factors. Here, we will focus on three subcategories based on our observation guide.

#### Objects

PAL involves the use of diverse objects, categorized as personal and public. Personal objects include items like notebooks and phones, while public objects encompass resources such as computers, electronic patient files, and large screens in educational rooms. Video (call) systems enable peer observation and review. Patients can also be seen as objects for investigation and learning, although PAL is less frequently employed in complex patient cases. However, objects can also have negative effects, such as presentation formats that hamper students' ability to follow the clinical-reasoning process, thereby hindering the learning experience.

#### Context

Not all contexts are equally utilized for PAL, despite their suitability. Contexts can vary in terms of location (inside or outside the hospital), privacy level (restricted/private or public spaces), and size (small or large rooms). Examples are found in Box 2.

The student working room holds particular significance, as participants recognize its potential but note its increasing disappearance in practice (I). This room is valued as a safe space for students to work without the presence of the supervisor who is assessing them (Q10) (I). Its absence results in students missing potential opportunities for collaboration.

**Box 2:** Examples of context where PAL takes place

- Extramural medical department - Outpatient clinic - Ward - Emergency department - Operating room - Physician working room - Student working room* - Education/report room - Secretariat - Break room - Hallways - Waiting room - Hospital cafeteria - Train - Car *A student working room is an, often enclosed, space at the workplace dedicated to students where they can speak freely while working	

The context in which students work plays a vital role in either facilitating or hindering PAL. When students are assigned to different workplaces within a department, they have less regular interaction, leading to decreased utilization of PAL. Some clerkship locations organize student meetings to foster a context particularly conducive to PAL. However, certain contexts are less supportive of PAL due to factors such as (1) limited space to accommodate multiple students, (2) strict work processes that leave less room for PAL (e.g., operating rooms or departments with vulnerable patients like pediatrics, emergency department, psychiatry) (I), or (3) shorter clerkship durations that pose time constraints on PAL implementation, as extensive learning needs to be accomplished within limited time frames (I). Participants consider wards to be excellent PAL contexts because of patients’ long stays (I).

#### Time

PAL's potential influence at the micro, meso, and macro levels is shaped by the factor of time. At the micro level, increased time spent together or dedicated to educational purposes fosters greater informal PAL engagement. However, rigid clerkship schedules that lack room for self-directedness often result in diminished peer interaction, leaving students feeling as if they are working individually despite being surrounded by others (I). Supervisors generally do not actively encourage students to utilize each other for their clinical-reasoning learning process, instead prioritizing individual patient encounters and, in fact, minimizing potential interaction among students (I). They suggest that students should take ownership of PAL and organize it themselves if they deem it necessary for their learning (I) (Q11).

At the meso level, numerous PAL opportunities arise throughout the day and are integrated into students' daily activities. These instances include discussions with supervisors after morning reports or during the mid-afternoon period when students have lighter workloads in a ward, allowing for peer interaction. Informal PAL occurs naturally at outpatient clinics while preparing for patient encounters, during joint lunch breaks or shared transportation to and from clerkship locations.

At the macro level, participants perceive the most PAL opportunities either at the beginning or the end of their Master's program (I). At the start, PAL can aid in faster acclimatization and guide students in developing clinical-reasoning skills in practice ("how it works in practice"). Towards the end of the program, students have developed their own styles, and discussions can delve into exploring differences and similarities in their practice, fostering deeper PAL interactions (I).

### The role of different actors present in the PAL-context

PAL moments involve the presence of several actors, most commonly (near-)peers, supervisors, and patients. These actors can either influence PAL or actively participate in PAL in specific roles.

#### Peers

Peers play a crucial role in facilitating the acquisition of clinical-reasoning skills through PAL. Students value their peers for their distinct perspectives, shared level of understanding, accessibility, approachability, and support (Q12) (I). Students often find that their peers comprehend their challenges better than physicians do (I). However, some students struggle to trust their peers' clinical-reasoning skills, as they perceive supervisors as the sole possessors of ultimate truth (Q13) (I). Nonetheless, most students can establish a safe learning environment with their peers, fostering vulnerability and growth, while there are a few who may experience minor competitive dynamics (Q4).

#### Near-peers

Near-peers, often senior students in their final year of undergraduate education, play a crucial role in practice settings, acting as intermediaries between supervisors and junior students. They have a better alignment with the cognitive abilities of junior students compared to supervisors and already possess advanced clinical-reasoning skills. These near-peers can serve as substitute supervisors until the clinical supervisor becomes available (I). Moreover, they serve as role models for junior students' future development. However, senior students often lack sufficient supervisory skills for junior students.

#### Supervisors

The combination of supervisors and PAL presents challenges. Supervisors are vital for content validation during PAL sessions about clinical reasoning, but students more often engage in PAL without their presence (Q14 and Q15). This creates a complex situation for supervisors, as participants express the need for guidance rather than authoritative leadership (Q16) (I). Effective guidance involves maintaining a background presence, intervening only when necessary, and directing questions back to students to encourage student-to-student discussions and deeper conversations (Q17). However, a common pitfall is supervisors answering questions themselves instead of returning them to the student group, resulting in limited student–student interaction (O). To initiate PAL, supervisors can facilitate connections between student groups and interesting patients (I). The competence to supervise the clinical-reasoning learning process is more important than the supervisor’s background (resident or medical specialist). Students adapt their presentations based on the audience, focusing on complexity with staff present and specific learning needs with student-only audiences (I).

#### Patients

Participants attribute various factors to patients in the context of PAL (I). Patient encounters are seen as the most effective way to develop clinical-reasoning skills (I). However, patients usually play a passive role and indirectly affect PAL. Students lower their voices around patients, driven by concerns about perceptions and fear of causing unnecessary worry. Supervisors may discourage PAL during patient encounters to avoid burdening patients with educating future healthcare professionals (I).

## Discussion

This research explored the manifestation of PAL in the clinical learning environment of undergraduate medical students with regard to developing clinical-reasoning skills. Students deploy various categories of PAL to advance their clinical-reasoning skills, although they were largely unaware of these processes, and facilitation of PAL is not consistently provided. Based on our results, we identified three topics of discussion regarding PAL and learning clinical reasoning, which are elaborated below.

The first topic concerns the environment. The learning context in which students need to develop their clinical-reasoning skills is often not conducive to self-directedness and collaborative learning with their peers (Noerholk & Tolsgaard, 2022). We observed that groups of students placed at a clerkship location are often allocated to different workplaces within the same location to ensure they do not hinder each other in their work. Concurrently, the context of modern-day clinical practice is adapted to future patient care, which also leads to changes in the student learning environment. The availability of the student working room, where students would typically meet each other, is often reduced, resulting in students mingling with residents and medical specialists. While this change can positively impact students' visibility within the community of practice (Lave, 1991; Lave and Wenger, 1991) and highlight their roles as apprentices to clinical supervisors, it has led to the removal of a dedicated meeting space. Such a space is crucial for enabling PAL discussions where students can freely exchange ideas without fear of judgment from their assessors (Lincoln & McAllister, 1993). Further research into the availability (or lack thereof) dedicated student working rooms could help us understand its role in the learning process of undergraduate students.

The second topic involves the role of peers in the clinical-reasoning learning process. In this research, we observed a reduced trust of some students in their peers’ (developing) expertise. The same-level peers they use for co-regulated learning during their Bachelor’s, are typically replaced with clinical supervisors during their Master’s program, as is also shown in other research (Bransen et al., 2020, 2022). This could be attributed to the fact that students are a part of a community of learners (Brown & Campione, 1990; Rogoff, 1994) during their Bachelor’s program, and that when they enter clinical practice they feel the need to take part in that specific community of practice with the medical specialist at the center (Lave, 1991; Lave and Wenger, 1991). Slowly, their community of learners disintegrates and merges into this community of practice, leaving the role of peers in a state of uncertainty. During their Masters’ program students develop new ways to use peers for their clinical-reasoning learning process. Whereas during their Bachelor’s program, students’ peer-to-peer contact moments were more formal and explicit (i.e. group assignments, team-based learning), they become more implicit and informal during their clinical clerkships (i.e. sparring partners). This research contributes to making these implicit PAL activities explicit, providing insights into the dynamics and opportunities to support students’ clinical-reasoning learning process in practice.

The third topic reflects an internal struggle for students. On the one hand, this generation of students applies a more individualistic approach towards learning in the workplace than those before them, not to be confused with a dislike for teamwork (Berkup, 2014; Bleakley, 2020). Although they acknowledge and value the role of their peers in their own clinical-reasoning learning processes, they prefer individual interactions with patients over those that occur together with a peer. This matches the anthropological view that people in the West are more individualistic than those in other parts of the world (Singelis et al., 1995; Strauss, 2000). Some respondents mentioned experiencing a sense of competition, which they believed could discourage their involvement in PAL. Since this individualism seems to be embedded into the students’ generation, more effort is needed for a change of behavior–a change that we deem necessary for a future of healthcare workers who are self-directed learners and excellent collaborators (Kemp et al., 2022), behavior that contributes to patient-centered care and arguably better clinical-reasoning skills (Tolsgaard et al., 2013). PAL could be of help in this behavior change.

Overall, these three topics describe important variables of students for either the success or failure of using PAL in the workplace for their clinical-reasoning development. Furthermore, they provide ample inspiration for practical implications and future research. Bringing a more collective awareness to the students’ individualistic behavior means changing the work-learning environment. PAL can be used to achieve these objectives, but in practice, is often presented as a formal learning activity. This adds to the prevailing tendency to structure students' learning formally around the daily activities of the community of practice, rather than organizing those activities in a way that fosters lifelong learning for all community members, including students (Clemans, 2015).

## Strengths and limitations

This research was conducted in The Netherlands, a country that is part of the Western world. Although more countries around the world are part of the same culture, it must be noted that these results possibly do not represent other areas of the world. This institute was conducted at one academic institute and its affiliated hospitals. This may be considered a limitation, although in one affiliated hospital students from another institute were included. Additionally, we only interviewed participants once and did not conduct follow-up interviews with the same participants to test our theories or their interpretations over time. We tested only the theories that were developed during the three-month data collection period, during which the analysis began concurrently. Lastly, there are certain forms of bias associated with this type of research that should be considered, like observer bias, attentional bias, and reporting bias.

One strength of this study concerns the methodology wherein all the data of the interviews and clinical observations were consistently collected and coded by two researchers. This initiated rich and deep discussions within the research group during analysis. Another notable strength of this study was the inclusion of two undergraduate students (SH, EV) as members of the research group. This helped in the validation of the interview guide and triangulation of the data. We found that focused ethnography is an elegant and time-efficient methodology for qualitative research questions that already have a particular focus and when there is an existing theoretical background on the phenomenon being investigated.

## Conclusion

This research demonstrates that students utilize each other to enhance various clinical-reasoning skills. Students deploy various categories of PAL to advance their clinical-reasoning skills, although they were largely unaware of these processes, and facilitation of PAL is not consistently provided. Together, they get further in the clinical-reasoning process, while subsequently learning from each other. This research positioned PAL theory alongside clinical practice, and its complexity is reflected in the results. Three topics are identified that need to be acknowledged: (1) the design of the PAL-environment, (2) the shifting roles of peers when they enter clinical practice, and (3) the individualistic tendencies of students. These topics serve as inspiration for future research, focusing on stimulating and facilitating PAL among the next generation of students and integrating PAL into the clinical practice workflow.

## Supplementary Information

Below is the link to the electronic supplementary material.

Supplementary file1 (DOCX 15 KB)

Supplementary file2 (DOCX 19 KB)

Supplementary file3 (DOCX 17 KB)

## Data Availability

A selection of the data is provided within the manuscript or supplementary information files. The comprehensive dataset that supports the findings of this study is not openly available due to reasons of sensitivity and is available from the corresponding author upon reasonable request. Data are located in controlled access data storage at Radboud University Medical Center.
