# Supplementary material for: It takes a village: an ethnographic study on how undergraduate medical students use each other to learn clinical reasoning in the workplace
Source: Adv Health Sci Educ Theory Pract. 2025 Feb 10;30(5):1453–71. doi: 10.1007/s10459-024-10404-5 (PMC12572093; doi:10.1007/s10459-024-10404-5)
Supplement: Supplementary file 1 — Supplementary file1 (DOCX 15 KB) [file 10459_2024_10404_MOESM1_ESM.docx]

**Appendix 1: Interviewguide PAL-research**

***NOTE:*** *These interview guides were originally in Dutch. This is a translated version.*

Main research question: What experiences are there with peer-assisted learning in the workplace for developing clinical-reasoning skills?

**Main questions for students**:

- At what moments do you encounter other medical students in the workplace?
- What do you do?
- What impact does it have?
- Who are involved? What roles do they have?
- What opportunities are there?

**Main questions for supervisors**:

- Have you ever witnessed a moment where medical students interacted with each other in the workplace?
- What were they doing?
- What role did you play in that? What role should a supervisor play?
- What opportunities are there?

**Topics:**

- PAL during formally organized education in the workplace
- Performing a task together with a peer
- PAL through peer-consulting/sparring
- Clinical reasoning

**PAL categories:**

- Peer modeling
- Peer assessment & feedback
- Peer mentoring/coaching
- Peer teaching
- Peer collaboration
- Peer monitoring
