# Supplementary material for: It takes a village: an ethnographic study on how undergraduate medical students use each other to learn clinical reasoning in the workplace
Source: Adv Health Sci Educ Theory Pract. 2025 Feb 10;30(5):1453–71. doi: 10.1007/s10459-024-10404-5 (PMC12572093; doi:10.1007/s10459-024-10404-5)
Supplement: Supplementary file 2 — Supplementary file2 (DOCX 19 KB) [file 10459_2024_10404_MOESM2_ESM.docx]

**Appendix 2 - Observation guide**

**NOTE:** This document was originally in Dutch. This is a translated version.

*Context*

1. **Space/environment**
   1. Location/space
      1. Reasoning behind choice of space
      2. Influence time/duration on choice of space
      3. Influence space on event/act/activity, participants, setting and achieving goals, feelings.
   2. Layout (i.e. of chairs/tables)
2. **Objects**
   1. Objects used to facilitate PAL
      1. Who use objects
      2. What are objects used for/How are objects used
      3. Impact of use of objects (on achievement of goals, on duration of PAL)
   2. Supply of objects in space (which can be used for PAL)
3. **Time**
   1. Time
      1. Reasoning behind choice of time for event
      2. Influence timing on event/act/activity or setting and achieving goals
      3. Influence other events on time
   2. Duration
      1. Reasoning behind choice of time period for event
      2. Influence time duration on event/act/activity or setting and achieving goals
      3. Influence other events on duration
4. **Actors**
   1. Who are involved in PAL?
      1. How do participants influence which PAL events are done?
      2. How do events influence who participates?
      3. How do the goals of PAL influence who participates?

*Acts/activities/events*

1. **Acts**
   1. Acts part of PAL/that lead to PAL (incl. who)
   2. Influence of event on what acts are done
2. **Activities**
   1. PAL activities (related to clinical reasoning)
   2. Influence of the event on what activities are done
3. **Events**
   1. PAL events (related to clinical reasoning)

*Emotions and experiences*

1. **Emotions and experiences**
   1. What feelings or experiences do actors express about PAL activities?
   2. Who are expressing these feelings or experiences?
   3. Where are these expressed?
   4. How are these expressed?

**Interview guide** *(for unstructured interviews during observations)*

These are sample questions designed to gather more information about specific activities that were observed, as well as the absence of anticipated activities or missed opportunities. The questions are optional and do not need to be posed for every observation.

1. PAL goals

- To what purpose did you do [PAL activity]? / What do you want to achieve with [PAL activity]?
  - Had you consciously thought about this goal?
  - Did you achieve that goal?
    - What made the goal (not) achieved? / Was there anything or anyone else influential in achieving the goal?
- Was there anything or anyone else influencing which goals you wanted to achieve?

1. Emotions and experiences

- How did you experience [PAL activity]? / What did you think of [PAL activity]?
  - What made you have this experience?
  - Did the space/time/actors affect you experience?
- What is good about [PAL activity]? Why?
- What could be improved about [PAL activity]? Why?
- What do you take away for yourself from [PAL activity]?
- Did [acts other actor] do anything to you? / What did you think of [acts other actor]?
  - Why did it do something to you?
  - Did the space/time/actor/activity affect how you experienced [PAL activity/acts other actor]?
  - How did you react to this? / Did you change your behavior as a result?

1. Missed opportunities

- Did you give any conscious thought on how you could make [event/activity] interactive?
  - If so: how? Can you give an example?
  - If not: why? As you hear this, would you like to do that? Do you have any ideas how you could do so?
- I saw [observation missed opportunity]. Did you give any more thought on this? / Was this intentional?
  - Do you have any idea why this was not done or did not happen? / What were the barriers to this not being done or occurring?
  - Would you like it if this were done/occurred? Why? Do you have any idea what it would take to make this happen?
