# Supplementary material for: It takes a village: an ethnographic study on how undergraduate medical students use each other to learn clinical reasoning in the workplace
Source: Adv Health Sci Educ Theory Pract. 2025 Feb 10;30(5):1453–71. doi: 10.1007/s10459-024-10404-5 (PMC12572093; doi:10.1007/s10459-024-10404-5)
Supplement: Supplementary file 3 — Supplementary file3 (DOCX 17 KB) [file 10459_2024_10404_MOESM3_ESM.docx]

| The role of oneself in PAL  The role of others in PAL  Student interactions in PAL | Mirror/compare | Giving feedback to peer or assess peer | Explain to peer |
| --- | --- | --- | --- |
|  | Engaged in learning process of peer | Gossip/vent | Observe |
|  | Sharing feedback with peer | Seeking or giving tips/advice |  |
|  | Learning from others’ experiences | Learning from each other’s patients afterwards | Help with research (where/how) |
|  | The other as an example/model | Receiving explanation from peer | Third person in peer interaction |
|  | Sparring | Discuss | Test each other |
|  | Share experiences with each other | (lack of) working together | Observe each other |
|  | Collaborate on presentation or education (preparation) | Prepare patient/case/disease together | Generate an answer together |
|  | Support | Empower | Coaching |
|  | Coach and learn from each other | Divide tasks and responsibilities | Perform physical examination together |
|  | Interaction | Do together | Learn together |

**Appendix 3: Open Coding of PAL-events**

***NOTE:*** *These codes were originally in Dutch. This is a translated version.*
